# Calibration of Correlation Radiometers Using Pseudo-Random Noise Signals

**DOI:** 10.3390/s90806131

**Published:** 2009-08-03

**Authors:** Isaac Ramos Pérez, Xavi Bosch-Lluis, Adriano Camps, Nereida Rodriguez Alvarez, Juan Fernando Marchán Hernandez, Enric Valencia Domènech, Carlos Vernich, Sonia de la Rosa, Sebastián Pantoja

**Affiliations:** 1 Remote Sensing Lab., Dept. Signal Theory and Communications, Universitat Politècnica de Catalunya (UPC) Campus Nord, Bldg D3, E-08034 Barcelona, Spain; E-Mails: xavier.bosch@tsc.upc.edu (X.B.-L.); camps@tsc.upc.edu (A.C.); nereida@tsc.upc.edu (N.R.A.); jfmarchan@tsc.upc.edu (J.F.M.H.); valenzia@tsc.upc.edu (E.V.D.); 2 DAS Photonics, Ciudad Politécnica de la Innovación, Camino de Vera s/n, Edificio 8F, E-46022 Valencia, Spain; E-Mails: sonia.delarosa@dasphotonics.com (S.de la R.); cvernich@dasphotonics.com (C.V.); spantoja@dasphotonics.com (S.P.)

**Keywords:** correlation radiometers, calibration, Pseudo-Random Noise, *PRN*

## Abstract

The calibration of correlation radiometers, and particularly aperture synthesis interferometric radiometers, is a critical issue to ensure their performance. Current calibration techniques are based on the measurement of the cross-correlation of receivers’ outputs when injecting noise from a common noise source requiring a very stable distribution network. For large interferometric radiometers this centralized noise injection approach is very complex from the point of view of mass, volume and phase/amplitude equalization. Distributed noise injection techniques have been proposed as a feasible alternative, but are unable to correct for the so-called “baseline errors” associated with the particular pair of receivers forming the baseline. In this work it is proposed the use of centralized Pseudo-Random Noise (*PRN*) signals to calibrate correlation radiometers. *PRN*s are sequences of symbols with a long repetition period that have a flat spectrum over a bandwidth which is determined by the symbol rate. Since their spectrum resembles that of thermal noise, they can be used to calibrate correlation radiometers. At the same time, since these sequences are deterministic, new calibration schemes can be envisaged, such as the correlation of each receiver’s output with a baseband local replica of the *PRN* sequence, as well as new distribution schemes of calibration signals. This work analyzes the general requirements and performance of using *PRN* sequences for the calibration of microwave correlation radiometers, and particularizes the study to a potential implementation in a large aperture synthesis radiometer using an optical distribution network.

## 1. Introduction

Synthetic aperture interferometric radiometers have been successfully used in radio-astronomy and more recently, they have been proposed for Earth observation as well. In radio-astronomy, due to the large antenna spacing, calibration is usually performed taking advantage of mathematical properties of the observables (cross-correlations between pairs of receiver outputs) [1]. However, in Earth observation, due to the wide field of view, the antennas must be closely spaced and have a very wide pattern, which increases mutual coupling effects. In addition, the magnitude of the observables decreases much faster with the antenna spacing and the signal-to-noise ratio rapidly degrades, preventing the application of redundant space calibration (*RSC)* or other techniques used in radio-astronomy [1,2]. *MIRAS*, the single payload of *ESA*’s *SMOS* mission [3], is the first synthetic aperture radiometer devoted to Earth observation. Its calibration is based on the injection of distributed noise as an alternative solution to alleviate the mass, volume and phase/amplitude equalization technological problems associated with the injection of centralized noise from a single noise source [4]. A similar approach has been implemented in other instruments, such as the Geostationary Synthetic Thinned Aperture Radiometer (*GeoSTAR*) [5], and mixed approaches with two-level noise injection plus *RSC* have been proposed for Geostationary Earth Orbit Atmospheric Sounder (*GAS*) [6].

Although distributed noise injection overcomes the technical challenges of centralized noise injection, it has also several limitations:

1. only separable errors, those can be assigned to each particular receiver, can be calibrated [7], and
2. the thermal noise introduced by the equalized distribution network itself introduces an error [8,9] that must be compensated by taking differential measurements acquired with two different noise levels.

Recently, arbitrary waveform generators have been used to generate controlled partially correlated noise calibration standards (*CNCS*) [10]. In this work, it is proposed the use of centralized Pseudo-Random Noise (*PRN*) sequences for calibration purposes. *PRN* signals are periodic signals with very long repetition periods that are used in a variety of applications, such as Code Division Multiple Access (*CDMA*) communications or Global Navigation Satellite Systems (*GNSS*). They have a relatively flat spectrum, resembling that of thermal noise, over a bandwidth determined by the symbol rate. The calibration of microwave correlation radiometers (either aperture synthesis, interferometric, or polarimetric) can benefit from these properties by replacing the noise sources by *PRN* generators. This approach has several advantages:

- the signal amplitude is constant, which allows higher receivers input power levels than in the case of injecting noise, without the need to allow a margin to avoid signal clipping. This makes the calibration less sensitive to the receivers’ thermal noise,
- all receivers are driven with the same PRN signal, which allows the calibration of baseline errors as well (baseline calibration refers to all errors associated to the particular pair of receivers forming a baseline, and not just the “separable” error terms that can be associated to each particular receiver) ,
- 1 bit/2 level digital correlators can be used, the same ones typically used for the noise signals to be measured later on,
- the signal pattern is deterministic and known, which allows new calibration strategies different from the cross-correlation between receivers’ outputs, such as the cross-correlation of receivers’ output with an exact replica of the input sequence,
- new approaches to distribute the calibration signal such as:
  - electrical distribution at baseband,
  - optical distribution with a modulation at RF followed by an opto-electrical conversion at each receiver input, or even
  - the generation of the calibration signal at each receiver’s input using a reference clock, and
- the PRN source can be turned ON for calibration and OFF during the measurements, without the thermal stabilization problems of noise sources. At the same time the isolation requirements of the input switch are fulfilled and EMC problems minimized.

In principle, other signals covering the whole receivers’ bandwidth could be used as well (chirp signals, etc.). However, in these cases the relationship between the measured correlation and the true one depends on the number of bits [11], and to increase the scale of integration of the correlators and reduce the power consumption, the number of bits is usually limited to 1 or 2 at most. This prevents using signals that do not behave as noise, unless the signal-to-noise ratio becomes too low.

This paper is organized as follows. First, the theoretical background and simulation description are introduced. Then, to validate the working principle, experimental results of the technique based on the Passive Advanced Unit for ocean monitoring (*PAU*) instrument [12,13] are presented. After that, a potential implementation using an optical fibre network is proposed to boost the advantages of this new calibration approach. Finally, a summary of the main conclusions of this work are discussed.

This innovative technique for calibrating microwave correlation radiometers can be applied as well to other communication systems or phased-arrays where the receiver’s frequency response needs to be measured with the system turned on.

## 2. Theoretical Basis and Simulator Description

One of the most important phases of the measurement acquisition using a correlation radiometer is the calculation of the so-called Fringe-Wash Function (*FWF*) [14]. It provides an estimate of the spatial decorrelation of the signals measured by the instrument due to the different paths toward the different antennas. Its phase and amplitude at the origin (*τ* = 0) are required to calibrate the correlation radiometer. In synthetic aperture interferometric radiometers, the shape of the *FWF* around *τ* = 0 is also used in the image reconstruction algorithms to compensate for the spatial decorrelation effects out of boresight. If receivers’ frequency responses are exactly the same, the *FWF* phase is equal to 0°, and its amplitude is equal to 1.

Considering a signal *x*(t) injected as input to the *i*-th receiver of a correlation radiometer, the output signal *y_i_*(t) will be function of the frequency response of the receiver itself *H_i_*(f); if the input signal spectrum covers the receiver’s bandwidth, it is then possible to retrieve *H_i_*(f) from *x*(*t*) and *y_i_*(t).

To avoid error amplification, the spectrum of *x*(t) must preferably be flat over the whole receiver’s band, generally thermal Gaussian noise is used in this kind of applications, but another type of signals that exhibit a flat spectrum over a given bandwidth are the *PRNs*, widely used in *CDMA* and *GNSS*.

The *FWF* of the baseline formed by channels *i* and *j* can be estimated from the normalized cross-correlation *ρ_ij_* [15] between the output signals *y_i_*(t) and *y_j_*(t). The correlation is calculated according to Equation (1), where *N* is the number of samples, and the result is normalized as shown in Equation (2):

(1) $$r_{y_{i}y_{j}}\left(m\right)=\frac{1}{N}\sum_{n=1}^{N}y_{i}\left(n\right)y_{j}\;\left(n-m\right)$$

(2) $$\rho_{\mathrm{ij}}\;\left(m\right)\hat{=}\frac{r_{y_{i}y_{j}}\;\left(m\right)}{{\left|r_{y_{i}y_{j}}\;\left(m\right)\right|}_{\mathrm{MAX}}}$$

It has to be pointed out that in the usual definition-, the *FWF* is normalized with respect to its value at the origin (*FWF_ij_* (*n*) = *ρ_ij_* (*n*)/|*ρ_ij_* (0)|), while in Equation (2), it is normalized with respect to its maximum value.

**Figure 1.**
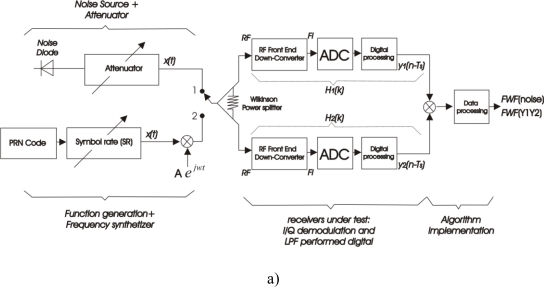

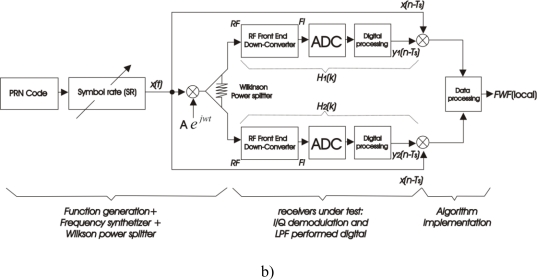
Block diagrams of the calibration approaches. a) *FWF*(noise) with the switch in position 1 and *FWF*(Y1·Y2) with the switch in position 2. b) *FWF*(local).

Two calibration methods are considered: injecting noise [*FWF*(noise)], as in Figure 1a with the switch in position 1, and injecting the *PRN* sequence [*FWF*(Y1·Y2)], as in Figure 1a with the switch in position 2. In both the cases several noise sources affect the result of Equation (1) such as the noise distribution network, the thermal noise present in *PRN* signal itself, leakages of the local oscillator noise through the mixer etc. All these contributions must be estimated and compensated for by taking differential measurements [9].

To overcome this problem *PRN* signals can be used to compute the receiver’s frequency response of the receivers before calculating the *FWF*. The receiver’s frequency response is computed through the correlation between a baseband replica of the *PRN* signal injected [*x*(n)] and the sampled output signals [Recall that the output signals are represented in complex form by their in-phase and quadrature components as: *y*(n) = *i*(n) + *j*·*q*(n)].

Being *y_i_*(n) = *h*(n) * *x*(n) + *n*(n), where *h*(n) is the discrete impulse response of the receiver and *n*(n) is a random noise term, and expressing the correlation between *x*(n) and *y_i_*(n) by Equation (3):

(3) $$r_{{\mathrm{xy}}_{i}}\;\left(m\right)=\frac{1}{N}\sum_{n=1}^{N}x\left(n\right)y_{i}\;\left(n-m\right)$$

The receiver’s frequency response can be calculated computing the Discrete Fourier Transform (*DFT*) of *R*_*xy*_*i*__:

(4) $$\begin{array}{l} R_{{\mathrm{xy}}_{i}}\;\left(k\right)\hat{=}\mathrm{DFT}\left[r_{{\mathrm{xy}}_{i}}\left(m\right)\right]=\mathrm{DFT}\left[x\left(m\right)\right]\mathrm{DF}T^{*}\left[y\left(m\right)\right]\\ =\mathrm{DFT}\left[\mathrm{PRN}\left(n\right)\right]\left\{\mathrm{DF}T^{*}\left[\mathrm{PRN}\left(n\right)\right]\mathrm{DF}T^{*}\left[h\left(n\right)\right]+\mathrm{DF}T^{*}\left[x\left(n\right)\right]\mathrm{DF}T^{*}\left[h\left(n\right)\right]\right\}\\ \hat{=}{\left|\mathrm{DFT}\left[\mathrm{PRN}\right]\right|}^{2}H^{*}\left(k\right) \end{array}$$

Isolating *H_i_* we obtain:

(5) $$H_{i}\;\left(k\right)=\frac{R_{{\mathrm{xy}}_{i}}^{*}\;\left(k\right)}{{\left|\mathrm{DFT}\left[\mathrm{PRN}\right]\right|}^{2}}$$

It has to be noticed that in (4) the correlation between *x*(n) and *n*(n) is zero.

Once the frequency response of the two channels involved *H_i_*(k) and *H_j_*(k) is determined, the *FWF* can be finally computed from:

(6) $$\Gamma_{\mathrm{ij}}\;\left(n\right)=\mathrm{IDFT}\left[H_{i}\;\left(k\right)\;H_{j}^{*}\;\left(k\right)\right]$$

(7) $$\rho_{\mathrm{ij}}\;\left(n\right)\hat{=}\frac{\Gamma_{\mathrm{ij}}\;\left(n\right)}{{\left|\Gamma_{\mathrm{ij}}\;\left(n\right)\right|}_{\mathrm{MAX}}}$$

where *IDFT* stands for the inverse *DFT*.

## 3. Experimental Validation of the Technique

The performance of the proposed technique has been assessed by measuring the *FWF* and its value at the origin in three different ways:

In order to compare and evaluate the performance of this technique, the first method, or ideal case, has been implemented injecting thermal noise [4] [“*FWF*(noise)”, as shown in Figure 1a, with the switch in the position 1]. The *FWF* is computed directly from the cross-correlation of the output signals of each channel using Equations (1) and (2).
In the second method [“*FWF*(Y1·Y2)”], the signal noise is replaced by a PRN signal (Figure 1a with the switch in the position 2). The *FWF* is also computed using Equations (1) and (2).
In the third method [“*FWF*(local)”, in Figure 1b] the output signal of each channel *y_i_*(n) and *y_j_* (n) is correlated with a local replica of the *PRN* (*x(*n*)]* to obtain *H_i_*(k) and *H_j_*(k) as in Equation (5). The *FWF* is then computed according to Equation (7). As additional feature, this method allows also to make a diagnosis of the receivers’ frequency response, which can be very helpful in monitoring the instrument’s health.

The *PRN* code is generated using a Linear Feedback Shift Register (*LFSR*) [16].

The selected length is 1,023 chips, which are recorded in an Agilent 33250A function generator, and upconverted using a Rodhe & Schwarz SMR40 frequency synthesizer.

The parameters that can impact the estimation of the *FWF* are:

1) the Symbol Rate (*SR*) defined as the ratio of the bandwidth of the *PRN* signal (*B_PRN_*) and the receiver’s low-pass equivalent bandwidth (*B*) [Equation (8)]. The *B_PRN_* is related to the sequence duration *τ_PRN_* and the number of chips (a chip is like a bit, but it does not carry any information) *N_chips_*, as shown in Equation (9):

(8) $$\mathrm{SR}=\frac{B_{\mathrm{PRN}}}{B}$$

and:

(9) $$B_{\mathrm{PRN}}=\frac{N_{\mathrm{chips}}}{\tau_{\mathrm{PRN}}}$$

**Figure 2.**
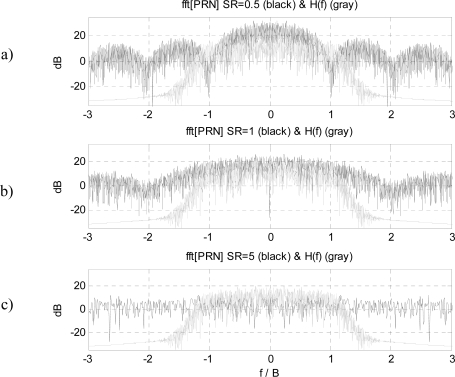
Equivalent low-pass spectrum of PRN sequence (black) with different Symbol Rates (SR) and H(f) estimated from noise (gray). Positive and negative frequencies plotted normalized to the bandwidth.

The higher the *SR*, the larger the bandwidth of the *PRN* signal spectrum, and the flatter is the spectrum within the receiver’s bandwidth (Figure 2). The minimum sampling frequency (*f_s_*) corresponds to one sample per chip *T_s_* = *1/B_PRN_*.

2) the equivalent noise temperature of the *PRN* signal (*T_PRN_*) at receivers’ input, defined in terms of the *PRN* signal’s amplitude (*A*) : *P_PRN_*=*A*^2^/2≙*k_B_*·*T_PRN_*·*B_PRN_,* where *P_PRN_* is the PRN signal power and *k_B_* is the Boltzmann constant (1.3806503 × 10^−23^ m^2^kg s^−2^K^−1^). The values of *T_PRN_* have been selected to be in the 6 K∼65,000 K range,

3) the number of averages. In fact since the *PRN* sequences are deterministic, averaging the measured Γ*_ij_* (*n*) values [Equation (6)], reduces the errors associated with the receiver’s thermal noise (*k_B_*·*T_R_*·*B*, being *T_R_* the receiver’s noise temperature). And,

4) the number quantization of bits.

Without lost of generality the described algorithms are tested using a PAU receiver [17] with the following parameters: gain *G* = 112 dB, noise figure *F* = 2.7 dB (*T_R_* = 250 K), RF bandwidth *B* = 2.2 MHz low-pass equivalent bandwidth= 1.1 MHz, central frequency *f_0_* = 1.57542 GHz, intermediate frequency *f_IF_* = 4.309 MHz. Results are presented normalized to the receiver’s bandwidth.

In this set-up the *SR* can be easily modified by reading the look-up table in the function generator at different speed. If the whole table is read in *τ_PRN_* = 1 ms and *B_PRN_* = *B*, then *SR* = 1 [Equations (8) and (9)]. The power level is adjusted with the frequency synthesizer. To minimize receiver’s noise, 200 consecutive *PRN* sequences are averaged, i.e. the integration time is *T_i_* = 200 *τ_PRN_*.

**Figure 3.**
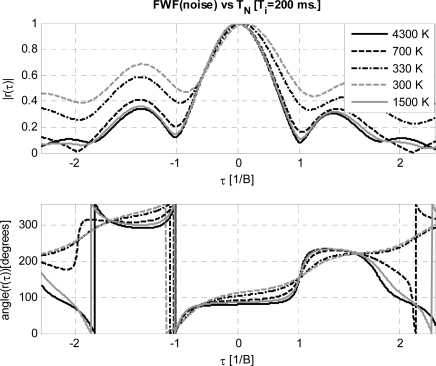
*FWF* estimated by cross-correlating receivers’ outputs at different time lags when injecting thermal noise at different equivalent noise temperatures *T_N_* [K].

In order to have a reference, Figure 3 shows the results of the *FWF*(noise) implemented with block diagram presented in Figure 1a with the switch in position 1, as a function of the input noise temperature *T_N_*. As *T_N_* approaches the physical temperature, the shape of the *FWF* degrades, since the noise introduced by the resistor in the Wilkinson power splitter, used to inject the noise to the two receiver chains, becomes comparable to the one injected (*T_N_*) and it is 180° out-of-phase in each branch-, leading to a zero cross-correlation. At too high *T_N_* the receiver saturates and clips the signal. The best results are obtained for *T_N_* / *T_R_* ranging between 2.7 and 16.7, and a value of 6 (*T_N_* = 1500 K) has been selected for all subsequent tests. Once the reference *FWF* has been determined, it is possible to analyze the *FWF* dependence on the three main parameters: *SR*, signal-to-noise ratio *SNR* = *P_in_/k_B_*·*T_R_*·*B* (≡ *T_PRN_/T_R,_* if *B* = *B_PRN_*) with *k_B_*·*T_R_*·*B =* −110 dBm*,* and the number of bits.

**Figure 4.**
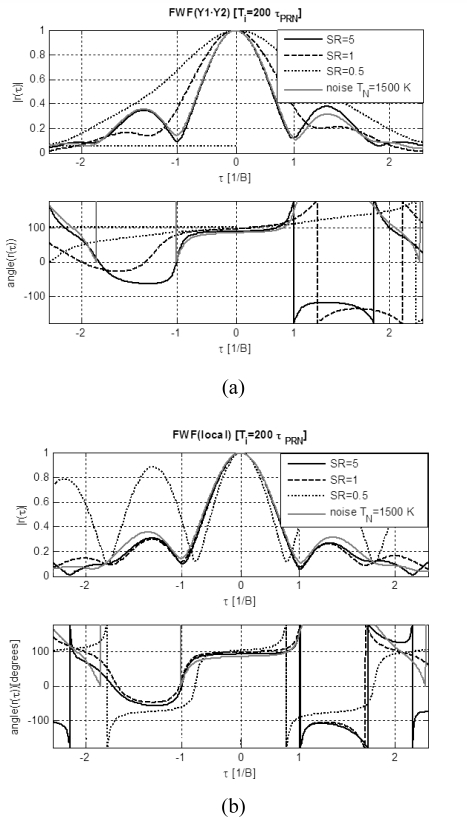
a) *FWF* estimated by cross-correlating receivers’ outputs when calibration signal is a *PRN* sequence *FWF*(Y1·Y2) (Equations 1–2) and comparison with reference *FWF* computed with correlated noise with *T_N_*= 1500 K (Figure 3). b) *FWF* estimated by cross-correlating receivers’ output with local replica of *PRN* sequence *FWF*(local) (Equations 3–7) and comparison with reference *FWF* computed with correlated noise with *T_N_*= 1500 K (Figure 3). Note: time axis is normalized to 1/*B.*

***FWF dependence on SR:*** To determine the optimum *SR* value a sweep has been performed for both *FWF*(Y1·Y2)] and (*FWF*(local) methods. Their performance has been analyzed and compared to the reference *FWF*(noise) (Figures 4a and 4b). It is found that for the *FWF*(local) method *SR* ≥ 1 is required to obtain a satisfactory *FWF*. The amplitude error does not improve significantly for *SR* > 1, but the phase error does, saturating above *SR* = 5. Slightly worse errors are obtained with the first method (*FWF*(Y1·Y2)] and higher *SR* values are required to obtain comparable residual error.

**Figure 5.**
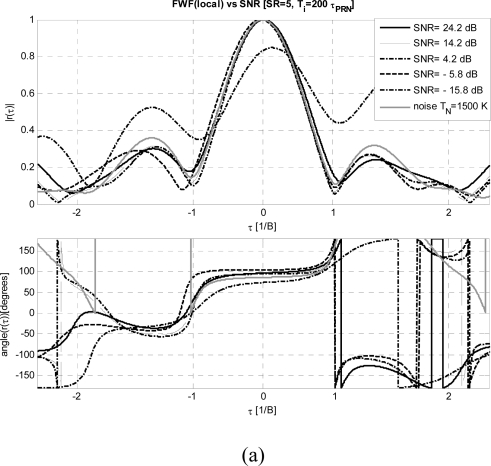

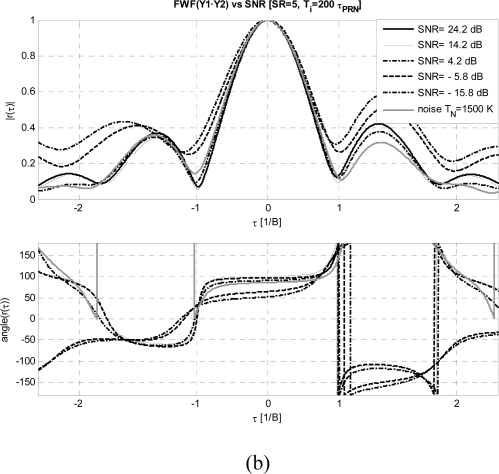
a) *FWF* estimated by cross-correlating receivers’ output with local replica of *PRN* sequence *FWF*(local) (Equation 3–7) for different input powers and comparison with reference *FWF* computed with correlated noise with *T_N_*= 1500 K (Figure 3). b) *FWF* estimated by cross-correlating receivers’ outputs when calibration signal is a *PRN* sequence *FWF*(Y1·Y2) (Equation 1–2) for different input powers and comparison with reference *FWF* computed with correlated noise with *T_N_*= 1500 K (Figure 3). Note: time axis normalized to 1/*B*.

**Figure 6.**
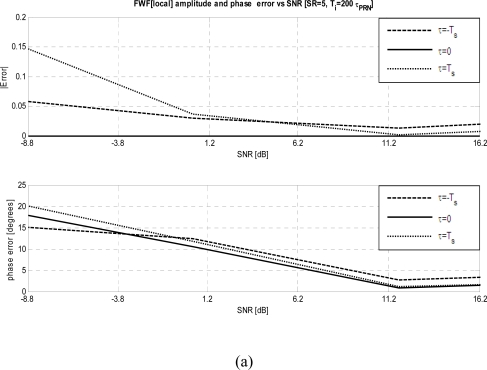

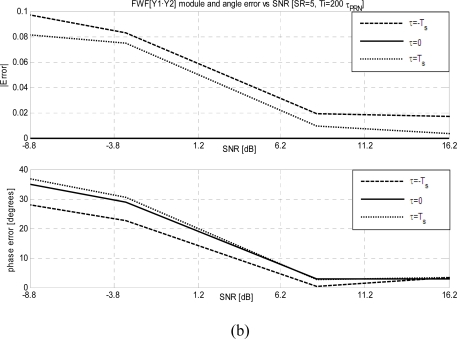
a) *FWF* amplitude and phase errors at τ = 0, ± *T_s_* when *FWF* is estimated by cross-correlating receivers’ output with local replica of *PRN* sequence *FWF*(local) (Equations 3–7) for different input powers. b) *FWF* amplitude and phase errors at τ = 0, ± *T_s_* when *FWF* is estimated by cross-correlating receivers’ outputs when calibration signal is a *PRN* sequence *FWF*(Y1·Y2) (Equations 1–2) for different input powers.

***FWF dependence on the signal input power:*** To determine the optimum power at receivers’ input, the input power has been swept while keeping *SR* = 5 and *T_i_* = 200·*τ_PRN_*. Except at the lowest input power, the *FWF*(local) method out performs the *FWF*(Y1·Y2) one (Figures 5a and 5b). Since the thermal noise present in the *PRN* signal being injected and the noise generated by the resistor of the Wilkinson power splitter, in fact, are completely uncorrelated with the local *PRN* sequence. In this case to retrieve the *FWF*(local) it is necessary at least that *SNR* ≥ +1 dB (Figure 5a) and optimum values (amplitude error < 2% and phase error < 5° at *τ* = ± *T_S_*) are obtained for *SNR* ≥ +11 dB (Figure 6a). For low input powers (*SNR* ≥ −9 dB) the amplitude errors using the *FWF*(Y1·Y2) method are smaller than using the *FWF*(local) one, while phase errors are twice higher. When the input power increases both methods provide similar results (Figure 6a and 6b).

**Figure 7.**
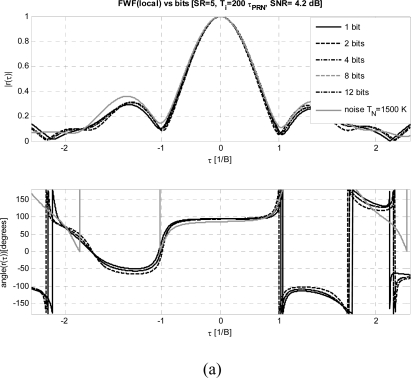

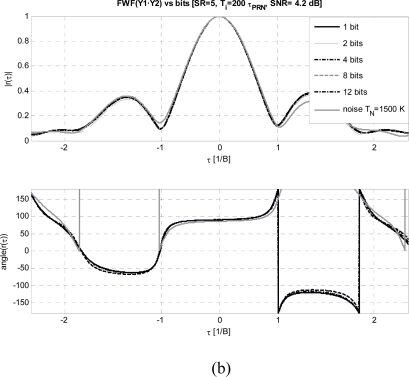
a) *FWF* estimated by cross-correlating receivers’ output with local replica of *PRN* sequence *FWF*(local) (Equations 3–7) for different number of quantization bits and comparison with reference *FWF* computed with correlated noise with *T_N_* = 1500 K (Figure 3). b) *FWF* estimated by cross-correlating receivers’ outputs when calibration signal is a *PRN* sequence *FWF*(Y1·Y2) (Equations 1–2) for different number of quantization bits and comparison with reference *FWF* computed with correlated noise with *T_N_* = 1500 K (Figure 3).

**Figure 8.**
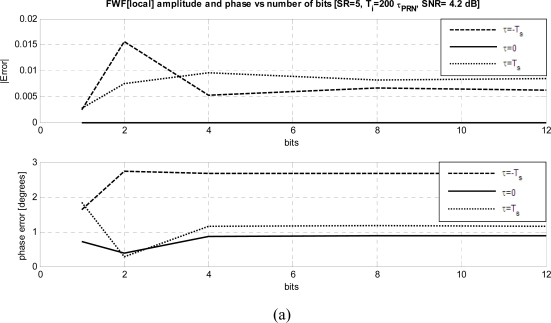

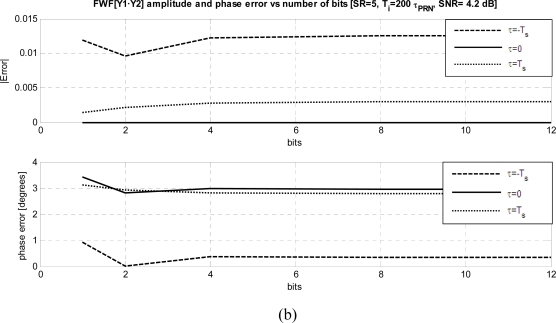
a) *FWF* amplitude and phase errors at τ = 0, ± *T_s_* as a function of the quantization bits when *FWF* is estimated by cross-correlating receivers’ output with local replica of *PRN* sequence *FWF*(local) (Equation 3–7), b) *FWF* amplitude and phase errors at τ = 0, ± *T_s_* as a function of the quantization bits when *FWF* is estimated by cross-correlating receivers’ outputs when calibration signal is a *PRN* sequence *FWF*(Y1·Y2) (Equations 1–2).

***FWF dependence on the number of bits:*** Figures 7a and 7b show the dependence of the estimated *FWF* as a function of the number of bits used to digitize the output signals from 1 to 12, while other parameters have been set to their optimum values: *SNR* ≥ +11 dB, *SR* = 5 and *T_i_* = 200·*τ_PRN_*. As it can be appreciated, as in other systems [18–20] there is a negligible variation with the number of bits above 4 bits for both methods, and very good performance is achieved even with just 1 bit. As it can be noticed, the residual errors especially for the phase, are much smaller with the *FWF*(local) method (Figure 8a), than with the *FWF*(Y1·Y2) one (Figure 8b).

As a summary, *PRN* signals can be successfully used to calibrate correlation radiometers. The best performance is achieved for *T_i_* at least 200·*τ_PRN_*, when the *PRN* signal bandwidth is about a factor 5 larger than the receiver’s bandwidth (*SR* ≥ 5) and the *SNR* ≥ +11 dB, even when one bit correlators are used. The optimum values using 1bit/2 level correlators, *SR* = 5, *SNR* = 4.2 dB and *T_i_* = 200 ms are: amplitude error < 0.25% at *τ* = 0, ± *T_s_*_,_ and phase error<1° at *τ* = 0 and <2° at *τ* = ± *T_s_*.

## 4. Considerations for the Implementation of a Calibration System for Large Aperture Synthesis Interferometric Radiometers

When dealing with the distribution of common *PRN* sequences to all receivers in a large aperture synthesis interferometric radiometer such as *MIRAS* in *SMOS*, *GeoSTAR*, *GAS*, etc. a number of different techniques can be devised:

1. Generation of the *PRN* signal in a central point and radio frequency (*RF*) distribution. In this case a distribution network similar to the current noise injection network would be required [4], or
2. Generation of the *PRN* signal in a central point and optical distribution to each receiver using an optical fibre distribution network, or
3. Generation of the *PRN* signal in a central point and baseband distribute it to all receivers. In this case an up-converter is needed at each receiver input, being all phase-locked to a common reference, or
4. Generation of the *PRN* signal at each receiver input. In this case, up-conversion, phase-locking and synchronism are required.

From the above four potential implementations, only the second one (centralized distribution using optical fibres), may provide a significant improvement over the current noise distribution system in terms of mass, volume and power while, at the same time, since all receivers will be driven with the same signal, it will provide a complete baseline calibration. It has to be noticed that the mass reduction associated with fibre distribution can be an enabling factor for future missions with higher number of receivers installed in a folding antenna.

For example, the current implementation of the *SMOS* mission includes the concept of optical signal distribution (*MOHA: MIRAS O*ptical *HA*rness), but it is limited to the distribution of digital signals of 122 Mbps between the receivers and the main data processor. The proposed alternative would complement this system with the optical distribution of the analog *PRN* signal as well. Three different alternatives could be envisaged:

With a different optical fibre than the one used in *MOHA* to send the sampled data to the correlator matrix located in the hub (#1),

With the same optical fibre used to send the sampled data to the correlator matrix located in the hub, using two different optical wavelengths (#2), or

Same as #2, but using the same optical wavelength and using optical directional couplers (#3).

Both options #2 and #3 need to add extra devices (Wavelength Division Multiplexers and optical splitters) that should be conveniently packaged to survive in space environment. These devices increase the mass and insertion losses of the distribution network, reducing the benefits of optical distribution. Option #1 needs the inclusion of a new optical fibre but as the lengths considered in satellite distribution systems are small the mass of the additional elements in #2 and #3 is higher than the corresponding to the extra fibre introduced in #1, being this option the optimum in terms of losses, mass, and reliability when compared to the other options.

**Figure 9.**
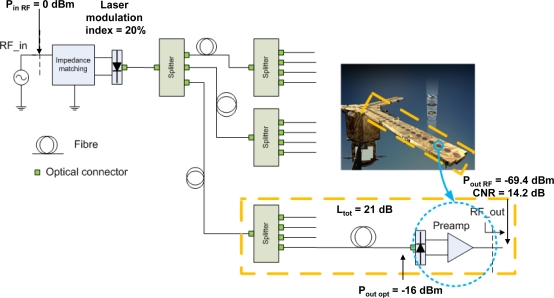
Concept block diagram of the implementation network in fibre optics [21].

Figure 9 shows a proposed *PRN*-based centralized calibration system based on option #1 [21]. The *PRN* signal is up-converted to the receivers’ central frequency *f*_0_ by directly modulating a laser, whose optical output is split to drive all the receivers. Then, at each receiver’s input, a photodiode detects the optical signals and converts it directly into an *RF* signal that is pre-amplified and injected in the receivers input where “correlated noise” is injected.

Optical signal distribution can find applications in satellite systems due to the possibility of mass savings that can be an enabling factor in some systems, especially in those with foldable antennas or high number of *RF* interconnects. Without loss of generality, the benefits of optical distribution of signals inside a satellite will be explored using the example of the system presented in this work, considering the optical distribution of the *PRN* signal to the receivers in the arms of the satellite.

For the particular case of *MIRAS*/*SMOS* the main parameters for the calculations are *B*∼22 MHz and *f*_0_ = 1.413 MHz and, since *SR* ≥ 5, the bandwidth of the *PRN* sequence must be larger than 110 MHz. In *MIRAS*/*SMOS* the total number of receivers is 69, but the *SMOS* follow-on missions may have even more. Therefore, to analyze the performance of this technique, without loss of generality, it is assumed that the total number of receivers is 100. Based on the results of [22], a preliminary design of the calibration system has been performed.

For the optical link shown in Figure 9, the link gain for a 100 element distribution with resistive impedance matching at the laser [23] is:

(10) $$G_{\mathrm{RF}}=\frac{1}{4}R^{2}\eta^{2}\frac{G_{\text{LNA}}}{L^{2}}$$

where *R* is the photodiode’s responsivity, *η* the laser’s emission efficiency (W/A), *G*_LNA_ the voltage gain of the receiver’s pre-amplifier, and *L* the optical distribution losses.

For simulation purposes, a medium power Distributed FeedBack (*DFB*) laser from Modulight, the same manufacturer as for the lasers used in *MOHA*, has been assumed in this example. It operates in the 1,550 nm wavelength and presents an efficiency of < 0.15W/A, with an output optical power <7 mW within the operational temperature range (from −20 °C to +85 °C). If a resistive impedance matching between the *RF* source (*P_in_* = 0 dBm) and the laser is used, and the distribution optical losses for 100 receivers (including connectors) are 21 dB, for an average emitted optical power of 3.75 dBm (and 40% modulation index to minimize harmonic distortion).

Since in the *SMOS* case, to neglect clipping effects, the maximum input power at receivers’ input is −83 dBm, the noise level is about −100 dBm, and the optimum *SNR* ≈ 11 dB, the calibration signal must be (P_ant RF_ = −69.4 dBm Figure 9) attenuated by ∼20 dB at the input of each receiver. With this approach the additional noise due to the optical distribution of the signals is kept below the receiver’s noise and thus does not degrade the system’s performance.

Regarding to the mass savings due to photonic distribution of the *PRN* signals, it has been assumed that a cable connects each antenna element in the arms with the body of the satellite, that is, a total number of 18 antennas x 3 arms = 54 links with a link length of roughly 2.2 m (distribution from arm center, see Figure 9 where arm length is 4 m plus 10% margin for cable deployment). Given these assumptions, the total mass for a copper distribution using Sub Miniature version A (*SMA*) connectors (3.8 g/unit) and 3 mm coaxial cable (26 g/m) is 3.5 kg, whereas optical distribution using miniature optical connectors (1.12 g/unit) and 1.2 mm simplex optical spaceflight cable (2.5 g/m) is 420 g, which results in more than 3 kg reduction (85%) in harness’ mass.

## 5. Conclusions

Correlation radiometers require the injection of known calibration signals. Currently these signals are generated by one or several noise sources and are distributed by a network of power splitters, which is bulky, difficult to equalize, and introduces additional noise. Aiming at alleviating these problems a new technique is presented. It consists of the centralized injection to all receivers of a deterministic *PRN* signal, providing complete baseline calibration. *PRN* signal exhibits a flat spectrum over the receivers’ bandwidth, which makes possible to use those for calibration purposes instead of the usual thermal noise. Since the *PRN* signals are deterministic and known, new calibration approaches are feasible:

1) through the correlation of the output signals at different time lags, as it is usually done when noise is injected, but allowing a much easier distribution of the signal to all the receivers simultaneously, or

2) through the correlation of the output signals with a local replica of the *PRN* signal, leading to the estimation of the receivers’ frequency responses and of the Fringe-Wash Function. In this last case the distribution network has no influence on the correlation coefficient, adding correlated noise.

This technique has been verified experimentally to assess its performance and the optimum parameters to be used. Excellent performance has been demonstrated by comparing the Fringe-Wash Function (*FWF*) shape, and the amplitude and phase values at *τ* = 0, ±*T_s_* to the ones obtained using the injection of two levels of correlated noise [4]. The optimum parameters are: integration time at least 200 times the length of the sequence (*τ_PRN_*), *PRN* bandwidth larger than 5 times the receiver’s bandwidth (*SR* ≥ 5), and the *SNR* = *P_in_/K_B_T_R_B* ≥ 11 dB. The number of bits used turned out to only slightly affect the results and even if one-bit correlators are used, negligible system performance degradation has been noticed. The optimum values are: amplitude error < 0.25 % at *τ* = 0, ±*T_s_*, and phase error < 1° at *τ* = 0 and < 2° at *τ* = ±*T_s_*. Increasing the integration time above 200 ms will reduces the effect of receivers’ noise in these estimates.

A preliminary design of the centralized distribution of *PRN* signals for very large aperture synthesis radiometers is presented using an optical fibre network. From the different possible topologies studied, the simplest and lightest uses an additional optical fibre to distribute the *PRN* signals.
