# Correction: Ramos-Pérez, I. *et al*. Calibration of Correlation Radiometers Using Pseudo-Random Noise Signals. *Sensors* 2009, *9*, 6131–6149

**DOI:** 10.3390/s90907430

**Published:** 2009-09-15

**Authors:** Isaac Ramos-Pérez, Xavi Bosch-Lluis, Adriano Camps, Nereida Rodriguez-Alvarez, Juan Fernando Marchán-Hernandez, Enric Valencia-Domènech, Carlos Vernich, Sonia de la Rosa, Sebastián Pantoja

**Affiliations:** 1 Remote Sensing Lab., Dept. Signal Theory and Communications, Universitat Politècnica de Catalunya (UPC) Campus Nord, Bldg D3, Tel.: +34-934-017-362, E-08034 Barcelona, Spain; E-Mails: xavier.bosch@tsc.upc.edu (X.B.-L.); camps@tsc.upc.edu (A.C.); nereida@tsc.upc.edu (N.R.A.); jfmarchan@tsc.upc.edu (J.F.M.H.); valenzia@tsc.upc.edu (E.V.D.); 2 DAS Photonics, Ciudad Politécnica de la Innovación, Camino de Vera s/n, Edificio 8F, E-46022 Valencia, Spain; E-Mails: sonia.delarosa@dasphotonics.com (S.de la R.); cvernich@dasphotonics.com (C.V.); spantoja@dasphotonics.com (S.P.)

It has come to our attention that in the original version of a paper published in *Sensors* recently [1], the names of four co-authors were misspelled with several hyphens missing. The names should be as indicated below:

Isaac Ramos-Pérez^1,^*, Xavi Bosch-Lluis^1^, Adriano Camps^1^, Nereida Rodriguez-Alvarez^1^, Juan Fernando Marchán-Hernandez^1^, Enric Valencia-Domènech^1^, Carlos Vernich^2^, Sonia de la Rosa^2^ and Sebastián Pantoja^2^
